# Dynamic Contact Angle Analysis of Protein Adsorption on Polysaccharide Multilayer's Films for Biomaterial Reendothelialization

**DOI:** 10.1155/2014/679031

**Published:** 2014-09-08

**Authors:** Safiya Benni, Thierry Avramoglou, Hanna Hlawaty, Laurence Mora

**Affiliations:** ^1^INSERM, U1148, LVTS, Institut Galilée, Université Paris 13, Sorbonne Paris Cité, 93430 Villetaneuse, France; ^2^INSERM, U1148, LVTS, UFR SMBH, Université Paris 13, Sorbonne Paris Cité, 74 rue Marcel Cachin, 93 000 Bobigny, France

## Abstract

Atherosclerosis is a major cardiovascular disease. One of the side effects is restenosis. The aim of this work was to study the coating of stents by dextran derivates based polyelectrolyte's multilayer (PEM) films in order to increase endothelialization of injured arterial wall after stent implantation. Films were composed with diethylaminoethyl dextran (DEAE) as polycation and dextran sulphate (DS) as polyanion. One film was composed with 4 bilayers of (DEAE-DS)_4_ and was labeled D−. The other film was the same as D− but with an added terminal layer of DEAE polycation: (DEAE-DS)_4_-DEAE (labeled D+). The dynamic adsorption/desorption of proteins on the films were characterized by dynamic contact angle (DCA) and atomic force microscopy (AFM). Human endothelial cell (HUVEC) adhesion and proliferation were quantified and correlated to protein adsorption analyzed by DCA for fibronectin, vitronectin, and bovine serum albumin (BSA). Our results showed that the endothelial cell response was optimal for films composed of DS as external layer. Fibronectin was found to be the only protein to exhibit a reversible change in conformation after desorption test. This behavior was only observed for (DEAE-DS)_4_ films. (DEAE-DS)_4_ films could enhance HUVEC proliferation in agreement with fibronectin ability to easily change from conformation.

## 1. Introduction

Real public health problem, atherosclerotic disease, is the first cause of death in industrialized countries [1]. The resulting treatments of cardiac ischemia use either drug treatments or techniques of myocardial reperfusion (bypass surgery or angioplasty). Over the past twenty years, percutaneous transluminal coronary angioplasty (PTCA), technical intervention in cardiology, took a place in the treatment of coronary stenosis ensuring blood circulation recovery for a stenotic coronary artery [1, 2].

Vascular tissue engineering aims to develop implantable substitutes with biological and biomechanical characteristics as close as possible to those of native vessels. The PTCA is a technique that allows interventional cardiology to overcome a stenotic lesion in a coronary artery (coronary angioplasty) [2]. Thus 90% of angioplasty includes the establishment of a stent, with an implantation rate around 1.5 stent/patient [2].

The implantation of biomaterials in the human body, even if they have a preventive or curative function as in the case of coronary stents, may cause undesired reactions, such as destruction of endothelial cells leading to thrombus or an inflammatory response [3]. The most common pathological reaction after stents implantation is restenosis. Many studies on the development of antithrombotic biomaterials have proliferated in recent years, due to the improvement of their biocompatibility by surface functionalization [3].

The aim of the present study is to develop stents coatings with polysaccharide polyelectrolyte films in order to optimize the reendothelialization of the stent after implantation in human vessels.

Surface material physicochemical properties such as chemical composition, roughness, wettability, charge, or viscosity affect cell adhesion [4–6]. Endothelial cell functions such as proliferation, differentiation, or apoptosis are directly related to their adhesion to the biomaterial. Cell adhesion is an essential phenomenon for survival [7]. In their physiological environment, endothelial cells are attached to extracellular matrix (ECM) proteins.* In vitro*, cell attachment is also mediated by adhesion proteins contained in the culture medium [8]. Thus, the ability of materials to adsorb adhesive serum proteins in a favorable conformation will determine their ability to induce cell adhesion and spreading [9]. In addition, it is known that cell adhesion and morphology influence their proliferation and differentiation [10]. Moreover, the surface chemistry has an impact on cell shape and growth. This highlights the importance of studying and controlling the surface of coronary stents to optimize their reendothelialization, which is based on protein adhesion to the substrate. Initial adsorption of a protein on a surface occurs very quickly and avoids the direct interaction between cells and the surface. This protein adsorption can be enhanced or inhibited by surface modifications, which involve proteins and surface hydration, interfacial charge redistribution, and proteins conformational changes. Characterization of protein adsorption on biomaterial surfaces is necessary to improve understanding of physiological phenomena and to guide the cellular response.

Multilayer films of polyelectrolytes (PEM) are now well known and often used in biomaterial functionalization [11] and polysaccharides have already been used to modulate protein adsorption [12]. The present work is focused on a dynamic analysis of protein adsorption on PEM polysaccharides films by dynamic contact angle (DCA).

Dextran is a biodegradable and biocompatible polysaccharide [13]. In contact with the physiological medium, dextran binds to erythrocytes and platelets and thereby increases their electronegativity, thus reducing the phenomenon of aggregation of erythrocytes [13]. Consequently, many studies have been performed with this polysaccharide.

We were focused in this study on derived molecules from dextran polycations and polyanions. In general, polycations are known to have physiological effects: they are antibacterial, antifungal, and antitumor [14]. Diethylaminoethyl dextran (DEAE-dextran) will be used as the polycation in the polyelectrolyte film. Indeed, at physiological pH, amine groups have a positive charge. DEAE-dextran is known to be harmless to the plasma membranes of cells at low concentrations [14].

Dextran sulphate was obtained by sulfating a region of dextran. It was the polyanion that was associated with DEAE-dextran to form the films. It has antithrombotic properties [3] and has been shown to have platelet anticoagulant properties [15].

The objective of this study was to build and characterize a multilayer film composed of dextran based polyelectrolytes to cover stents in order to promote reendothelialization following implantation of the stent into the coronary arteries. For this, the dynamic of protein adsorption phenomena on the multilayer films has been analyzed by DCA, and the relation with the endothelial cell response was observed. Films have been constructed on square glass samples with a minimal roughness (Ra < 3 nm) to exclude this parameter that modulates contact angle values. Thus, only chemistry and charge of the surface will be considered to be the major parameters having an effect on protein adsorption and conformation in this study.

DCA is a sensitive technique to measure dynamic changes in wetting tension. It gives information on dynamic interfacial changes at interface between biomaterials and biological mediums. It brings information on the conformation of proteins adsorbed on biomaterial surface and on the reversible character of this conformation [16].

## 2. Material and Methods

### 2.1. Polysaccharide Polyelectrolyte's Films

**Figure 1:**
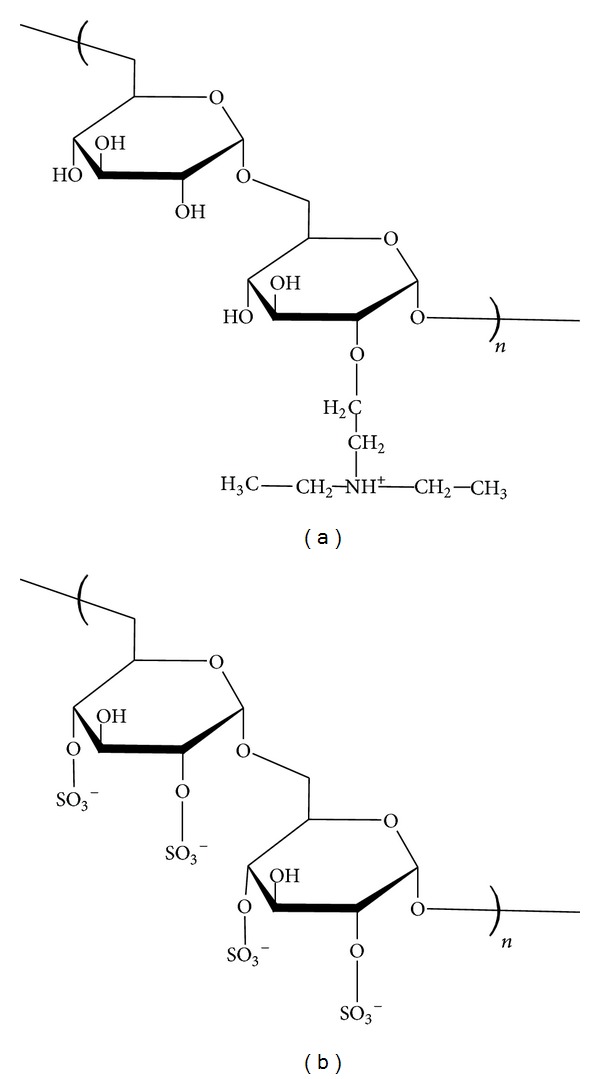
DEAE-dextran (a) and dextran sulphate (b).

Polyelectrolytes selected for this study were derived from dextran polysaccharides. The polycation was diethylaminoethyl-dextran hydrochloride (DEAE) and the polyanion was dextran sulphate (DS), both purchased from Sigma-Aldrich, UK, and with a mass of 500 kDa (Figure 1).

Polyelectrolyte multilayer films were deposited on glass slides (20 × 20 mm, VWR) that were cleaned in order to create negative charges at their surfaces: they were first immersed in a 1 M NaOH solution heated at 90°C for 30 min and then in a 1 M solution of HCl at room temperature for 10 min. All solutions were prepared with ultrapure water Millipore (18.2 MΩ/cm). Slides were then stored in ultrapure water. Polyelectrolyte's solutions were prepared at a concentration of 5 mg/mL in a 0.15 M NaCl solution at physiological pH. Slides were dipped in baths containing alternatively each polyelectrolyte for 10 min and 4 bilayers were finally deposed before a final rinsing in saline solution for 3 min. Two films resulted from this preparation, one with the outer monolayer of polyanion DS (negatively charged) that was labeled D− instead of (DEAE-DS)_4_ and the other one with a supplementary polycation DEAE-dextran layer added as the terminal layer (positively charged) that was labeled D+ instead of (DEAE-DS)_4_-DEAE, to simplify the notation.

### 2.2. Dynamic Contact Angle Measurements by Wilhelmy Plate Method: Protein Adsorption/Desorption

#### 2.2.1. Surface Characterization

Tensiometry measurements were performed using a Wilhelmy balance tensiometer fitted with a computer module for contact angle and sorption analysis (K100MK2 from Krüss Gmbh). Theory of this method is described elsewhere [17]. Contact angle is related to surface roughness and chemical heterogeneities (thermodynamic hysteresis). The evolution of contact angles with numerous successive cycles (during time) indicates molecule's motility or reorientation and swelling (kinetic hysteresis). In this study, 10 successive cycles were registered for each experiment: 5 cycles of wetting/dewetting for protein adsorption and few seconds just after and directly 5 cycles of wetting/dewetting in PBS solution for rinsing (desorption test). Wetting/dewetting rate in ultrapure water was 12 mm/s and immersion depth was 10 mm. Before each measurement, films were rinsed in ultrapure water 30 min in order to eliminate residual salts of NaCl and finally dried 3 h at 37°C as a reference surface before DCA measurements.

#### 2.2.2. Protein Adsorption/Desorption 

Protein solutions were prepared in NaCl 90% (Sigma-Aldrich, UK, 2 mg/mL). All the proteins, fibronectin, vitronectin, and bovine serum albumin (Sigma-Aldrich, UK), were prepared at 0.2% of total plasma proteins corresponding to physiological concentration (fibronectin at 1 *μ*g/mL, vitronectin at 0.6 *μ*g/mL, and BSA at 70 *μ*g/mL). Tensiometry experiments were conducted at room temperature and during 5 loops for adsorption followed by 5 loops in PBS for protein rinsing/reorientation test. All loops were measured at immersion and emersion rates 6 mm/min and the immersion depth was 10 mm. Extrapolated force, calculated by linear regression to zero immersion (to eliminate buoyancy force), was used as characteristic parameter here instead of contact angle because surface tension of protein solution is modified from one cycle to another and is no more constant during the overall series of cycle's acquisition.

### 2.3. Atomic Force Microscopy (AFM)

Atomic force microscopy (Nanoscope III Digital Instruments Dimension 3100) was used to image the surfaces of the two types of film. Theses surfaces were investigated in tapping mode for a morphological analysis surface of 10 × 10 *μ*m^2^. Topographical and phase images were registered and average roughness values (Ra) could be extracted by calculation using the Nanoscope Analysis Version 1.40r1 software.

### 2.4. Cell Culture: Adhesion and Proliferation Tests

Polyelectrolyte's multilayer (PEM) films were prepared from filtered polyelectrolyte solutions and ultrapure water. Samples were rinsed in ultrapure water to remove the salt (0.15 M NaCl) and then dried in an oven at 37°C for one hour. They were then sterilized for 15 min on each side under UV radiation at 254 nm.

To perform the culture of human umbilical vein endothelial cells (HUVECs, N° CRL-1730, from ATCC) on polyelectrolyte films, the samples were conditioned beforehand. One day before the inoculation, the PEM samples were placed in 6-well plates (6-well cell culture cluster, tissue culture polystyrene, sterile, Corning Incorporated, Costar, NY USA), on titanium holders. The samples were then incubated in complete culture medium (Endothelial Cell Basal Media 2; PromoCell, Heidelberg, Germany), supplemented with 10% of fetal calf serum (Lonza, Basel, Switzerland) and a mix solution from PromoCell. The HUVECs were detached from culture dish and collected in a 10 mL cell suspension solution. The samples were placed in the bottom of 6-well culture plate and 2 × 10^4^ cells/mL of HUVECs was added (cell counting by using a Coulter Counter ZM). The volume reaches 500 *μ*L with complete culture medium (DMEM, Gibco by Life Technologies). All PEM samples were then incubated in 37°C with 5% CO_2_ and H_2_O saturated.

#### 2.4.1. Cell Adhesion

Cell adhesion was assessed by a test of MTT (3-(4,5-dimethylthiazol-2-yl)-2,5-diphenyl tetrazolium). The quantification is performed in a drive space (Absorbance Microplate Reader BIOTEK) through Gen5 by measuring the optical density (OD) at a wavelength of 540 nm software.

#### 2.4.2. Cell Proliferation

Cell proliferation was performed for six days and cell number per well was counted every day, after incubation at 37°C. The cells are counted using the particle counter (Coulter Counter ZM, Coultronics).

### 2.5. Cell Response by Fluorescence Microscopy

HUVECs were observed using a fluorescence microscope (Zeiss Axiophot, Carl Zeiss, France) at ×100 magnification 48 h after incubation on glass, D−, and D+ samples. The observation of the cell shapes and cytoskeleton was performed with the fluorescent marker Alexa Fluor 546 phalloidin (F-actin/cytoskeleton, dilution 1/100, Invitrogen).

### 2.6. Statistical Analyses

Measurements of DCA contact angles and wetting tensions were conducted five times for each sample. We also took five samples of each for the MTT test. To study proliferation we took three samples for each surface and performed the experiment separately three times. Significant differences were affirmed by the Student's *t*-test, with a threshold of *P* = 5% for DCA measurements and cell viability and *P* = 0.001 for cell proliferation.

## 3. Results and Discussion

### 3.1. Films Characterization by DCA and AFM

In order to avoid any impact of roughness on contact angles measurements, the polyelectrolyte's multilayer (PEM) films were deposited on glass slides exhibiting practically no roughness (Ra < 3 nm). The specific chemical treatment was performed on the glass surface to exhibit the negative charges on it and facilitate the better anchorage of the first positively charged layer of the film [18]. This treatment leads to an advancing contact angle at 50° measured by DCA (tensiometer) due to silanol groups (Si–OH) which is slightly more hydrophobic than glass cleaned with a simple piranha (H_2_SO_4_/H_2_O_2_ 7 : 3) solution (43° by tensiometry), whereas it reached 50° by captive bubble method and 10° by sessile drop method [17]. Thus, contact angles can exhibit clearly different values depending on the surface cleaning process and on the method used for these measurements.

In order to ensure the total recovering of glass surfaces, five bilayers were deposited to construct the films [19]. Physicochemical properties of PEM films can be controlled with changing the charge of external layer [20]. Moreover, surface charge of biomaterials can modulate protein adsorption [3] and cell adhesion [6]. The aim of this study was to compare two polysaccharides, as terminal layer of the film, with close chemical properties but with two opposite charges.

**Figure 2:**
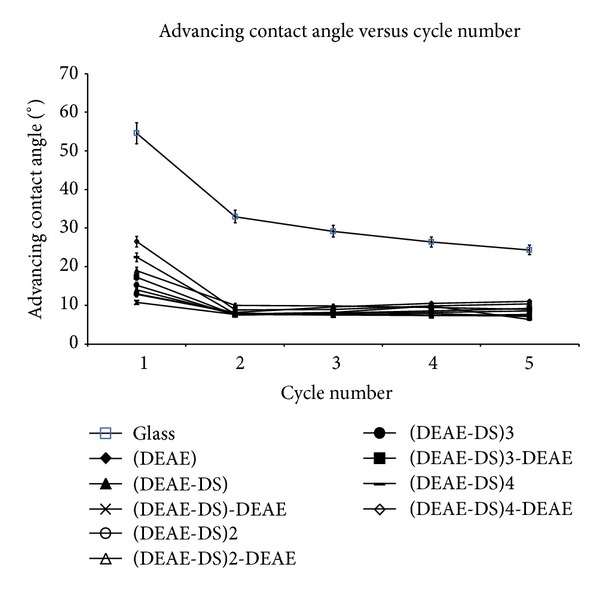
Advancing contact angle versus wetting/dewetting cycles.

The evolution of advancing contact angles as a function of cycles was represented on Figure 2. Each step of PEM elaboration was compared to glass. The surfaces of D+ and D− PEM final films were significantly more hydrophilic than surface of glass (*P* < 0.05). There was a significant decrease of contact angle in each cycle for all samples. More precisely, a strong kinetic hysteresis was observed, particularly between the first and the second cycles (swelling, water retention, macromolecular reorientation, or partial resolubilization-desorption). In addition, the surfaces of D− were also significantly more hydrophilic than D+ (*P* < 0.05).

**Figure 3:**
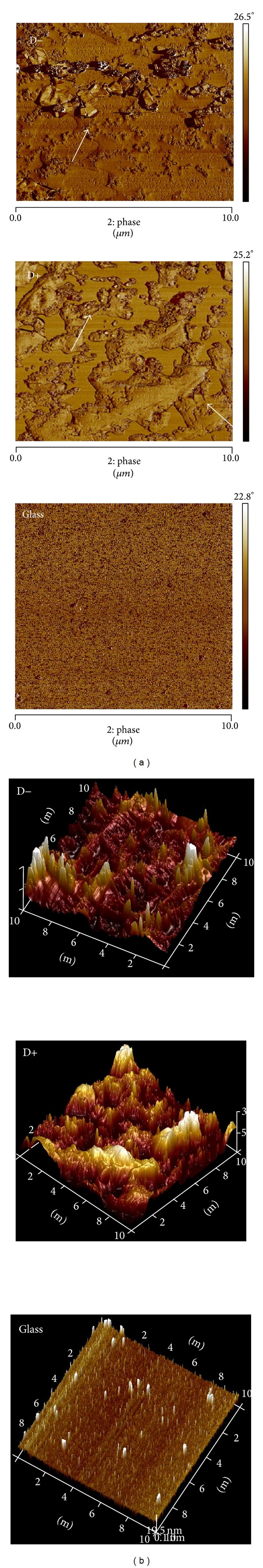
AFM images (10 *μ*m × 10 *μ*m) of D− (1), D+ (2), and glass (3) surfaces: phase (a) and 3D topography (b).

In the second step of our work, we analyzed the surface roughness of our layers D+ and D− using AFM analysis (Figure 3). Corresponding average roughness was found to be 72 nm for D+, 47 nm for D−, and 3 nm for glass control. The PEM films did not have the same topographical morphology as that of glass control, confirming that in presence of over 4 bilayers, the original glass was well covered by PEM and it was not visible anymore. Indeed, the surface roughness of D+ and D− layers appeared clearly as compared with the particularly smooth glass and the clusters or the granules, indicated by arrows, corresponding to local concentrations of polyelectrolytes. In addition, these clusters were more pronounced for the D+ layer than for D−.

### 3.2. Dynamic Protein Adsorption by DCA and Cell Response

#### 3.2.1. Dynamic Protein Adsorption by DCA

In the next step of our work, we studied the protein interactions with the PEM surfaces using DCA analysis. Proteins absorbed on the surface can migrate freely towards these surfaces and the information of surface coverage and degree of adsorption reversibility can be obtained with DCA, measuring the entropic effects such as modifications of adsorbed protein conformation and hydrophobic effect [21].

The three proteins used in this study, bovine serum albumin (BSA), fibronectin, and vitronectin, are major plasma proteins. BSA is the most abundant serum protein. BSA migrates at the same speed as that of other mammals, towards the anode when electrophoresis is carried out. BSA is therefore used as a model protein for albumin. It is a globular protein of 67 kDa whose isoelectric point is between 4.5 and 4.7. It is present in a plasma concentration from 35 to 50 g/L. This protein is most concentrated in the plasma since it accounts for 60% of the plasma proteins.

The plasma proteins fibronectin and vitronectin are known to interact with the surface of biomaterials quickly after implantation. Moreover, they are responsible for cell attachment to a substrate by providing a first anchor and adapting their three-dimensional structure. Fibronectin (Fn) is a large glycoprotein of 440 kDa, consisting of two similar subunits of 220 and 250 kDa. Its isoelectric point is 5.0. In the plasma, it is in soluble globular form (plasma concentration is 300–400 *μ*g/mL) while it is in insoluble fibrillar form in the extracellular matrix (ECM). Thanks to its many membership sites, it modulates cell adhesion. Fn plays an important role in cell adhesion by binding to membrane receptors via a pattern of three amino acids (arginine-glycine-aspartic acid), called RGD sequence. With this sequence, Fn participates in the control of a number of cellular processes such as cytoskeletal organization, proliferation, and differentiation.

Vitronectin (Vn) is an abundant plasma glycoprotein of 75 kDa, which is also found in the extracellular matrix. Vn is present in plasma (plasma concentration is 200–400 *μ*g/mL) and thus represents 0.2 to 0.5% of total plasma proteins. Vn has a single protein chain or two combined channels. Its isoelectric point is between 4.75 and 5.25. It promotes cell adhesion and interacts with complement, coagulation, and fibrinolysis proteins.

**Figure 4:**
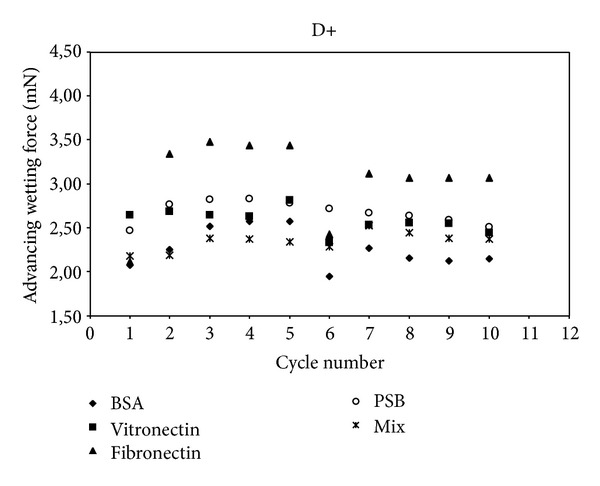
Advancing wetting tension (mN) as a function of wetting/dewetting cycles for D+ surface and for bovine serum albumin (BSA) vitronectin, fibronectin, and a mixture (Mix) of the three proteins. PBS is corresponding to D+ in PBS without any protein (reference curve).

**Figure 5:**
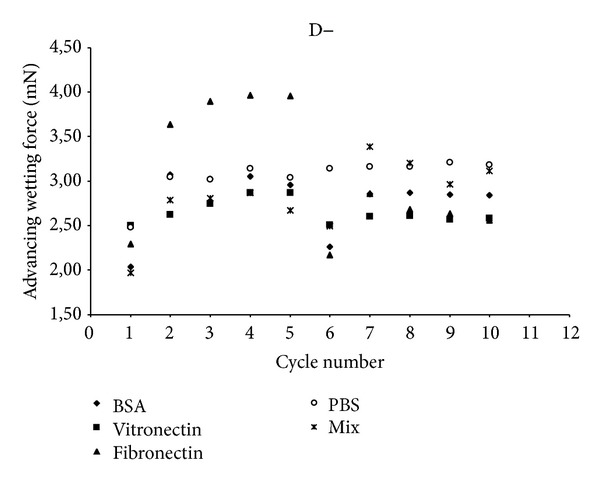
Advancing wetting tension (mN) as a function of wetting/dewetting cycles for D− surface and for bovine serum albumin (BSA) vitronectin, fibronectin, and a mixture (Mix) of the three proteins. PBS is corresponding to D− in PBS without any protein (reference curve).

**Figure 6:**
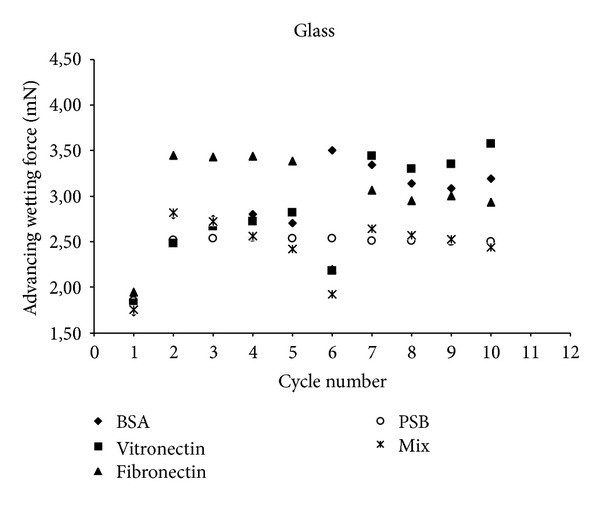
Advancing wetting tension (mN) as a function of wetting/dewetting cycles for glass surface and for bovine serum albumin (BSA) vitronectin, fibronectin, and a mixture (Mix) of the three proteins. PBS is corresponding to glass in PBS without any protein (reference curve).

In this study, we used 0.2% of physiological plasma concentrations of each protein [22]. The proteins were prepared in solutions of 90% NaCl at 2 mg/mL. We also studied the cycles of adsorption and rinsing from a mixture of these three proteins. These experimental conditions were prepared in accordance with the physiological proportions to approximate physiological conditions (named “Mix”). Then, the evolution of wetting tension F versus wetting/dewetting cycles was analyzed for D+, D−, and glass control (C1 to C5 for wetting adsorption/dewetting and C6 to C10 for wetting rinsing in PBS (reorientation)/dewetting, Figures 4, 5, and 6). This rinsing step destroys interphase organization [23] and removes not strongly bounded proteins from the surface. In this work, we tested and evaluated this interfacial rinsing by DCA, since its efficiency is still not clearly tested and explained in the literature [24]. We used the dipping solutions containing BSA, Fn, Vn, or a mixture of these 3 proteins (Mix). The curve of the corresponding surface in PBS without any protein was drawn as a reference control without any adsorbed protein at its surface. The wetting tension increased and reached a stationary steady-state plateau after the second cycle C2. During the first cycle (C1), the analyzed interactions were protein/polyelectrolyte, whereas they were protein/protein for the following cycles (C2 to C5). The same behavior was generally observed in the rinsing phase after cycle 6 with stabilization up to cycle 7. There were phenomena of competition between all proteins for joining the multilayer film to achieve a balance resulting in a gradual stabilization of the value of wetting tension over the cycles, for both adsorption and rinsing steps. Wetting tension steady state in the rinsing step could be increased, decreased, or not significantly modified compared to adsorption steady stat. It could approach PBS line or go away far from the line, remaining in the same side of PBS line (up or under the PBS reference wetting line) or crossing it, depending on surface/protein couples. If the changes in these two steady states were significant, it was probably that protein adsorption was reversible and that proteins conformation/orientation/quantity could be modified by the rinsing step.

Interestingly, our results indicated that only one protein Fn absorbed on only one surface D− showed a crossing of PBS line between adsorption and rinsing steps. This evidenced a strong change in protein conformation (*P* < 0.05) with a very flexible behavior qualified as reversible conformation. In addition, Fn was much more hydrophilic in adsorbed configuration than in rinsed one since wetting force after rinsing was strongly lower than before rinsing. All the other proteins remained in the same side of PBS line when rinsing occurred after adsorption. Thus, the couple Fn/  D− had an exceptional behavior in DCA measurements, compared to all the other proteins/surface couples. Fn was particularly flexible and able to reorient during rinsing step, on D−. Thus, DCA was a very sensitive tool to evaluate protein adsorption reversibility. It was not the case for D+ surface. All other single proteins attached on all surfaces (D+ and D−) exhibited nonreversible adsorption (*P* > 0.05).

In another hand, for glass-control surface, all single proteins remained at high wetting forces, with a high hydrophilic conformation, enhanced by rinsing step. This was due to the fact that glass is the most hydrophobic surface in this study. All proteins could interact with the glass, since they had the hydrophobic interactions of their nonpolar groups, exhibiting thus their polar groups towards the water based proteins solution (high wetting forces). It has been previously shown using scanning force spectroscopy to study the protein adhesion on dental surfaces that adhesion forces should be correlated to DCA surface coverage by BSA since no hydrophobic surfaces or covalent bonds were involved in this adsorption process [21].

Actually, glass cannot really be compared to D+ and D− surfaces, since D+ and D− carry strong Lewis acid/base functional groups with ion-exchange properties and can adsorb proteins using ion-exchange mechanisms that are not possible with glass surface [25].

Mix proteins returned systematically towards PBS line in rinsing step, for all the surfaces, PEM surfaces, and glass. Mixing proteins during adsorption process induces proteins/proteins interactions as well as proteins/surface interactions. Thus, the mix proteins/surface interactions should be diminished/modified, as compared to the conditions with single protein adsorption. The adsorption process is not as binary as for single protein adsorption and mix protein did not clearly increase (or decrease) the hydrophobicity of the initial surface. The final surface modification was very low and wetting force was not significantly changed, as compared to initial surface.

**Figure 7:**
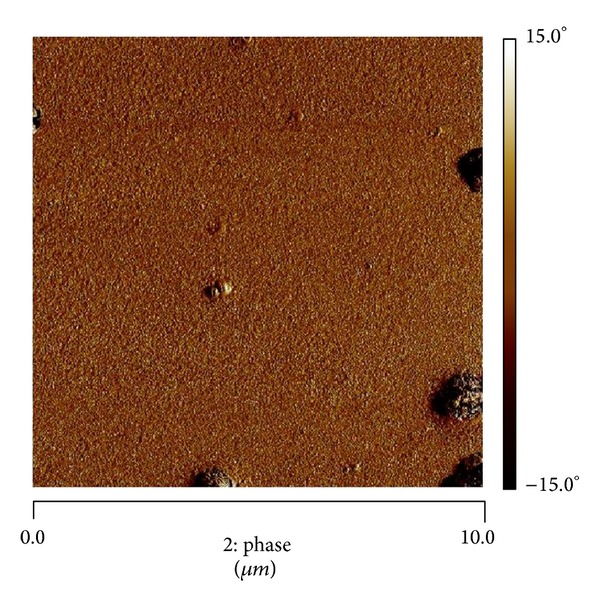
AFM in phase mode of D−/BSA after 5 cycles (cycle number = 5).

The next step of our work was to quantify the protein conformation reversibility between adsorption and rinsing steps, by comparing the wetting tension average values for these two steady states. There was only one protein, Fn, that showed a significant conformation reversibility during adsorption and rinsing steps. All other single and mix proteins could thus be qualified as irreversibly adsorbed. The reversibility of Fn conformation cannot be related only to a partial/total protein desorption during rinsing step but also to protein reorientation as long as the wetting force do not join the PBS line. This represents typically DCA kinetic hysteresis, by opposition to thermodynamic hysteresis due to roughness and/or chemical heterogeneities [22]. Anyway, even if the wetting force joins the PBS line, it is not an evidence that total desorption has occurred because wetting tension values are representatives of a convolution of the quantity and the conformation of the proteins adsorbed to the surface. Indeed, the same wetting tension value can be reached for two different surfaces, depending on the presence or the absence of proteins and on their corresponding orientation and hydrophobicity. Joining PBS line is a necessary but not sufficient condition to guaranty total protein desorption. This was evidenced by AFM analysis for a few couple's proteins/surfaces, for BSA adsorbed onto D− surface at cycle 5 (Figure 7). In addition, our results showed that wetting force at this moment was very close to PBS surface reference line (Figure 5). One explanation is possible that no proteins were present at this stage of the experiment; however, AFM images clearly showed the reverse and Ra (0.65 nm) was also strongly diminished compared to D− without proteins (Ra = 54.7 nm). Indeed, BSA deposition could smooth initial D− roughness. The same conclusion can be brought for Vn/  D− with a decrease to Ra = 32.2 nm. Similarly, Ra = 12 nm for Vn/D+, whereas Ra = 71.9 nm for D+ with no proteins, and the wetting forces values are superposed (Figure 4). Finally, there was Ra = 85.7 nm for Vn/Glass, whereas there was Ra = 31.6 nm for glass without proteins, as compared to PBS, and wetting forces were not so different (Figure 6).

#### 3.2.2. Cell Response

**Figure 8:**
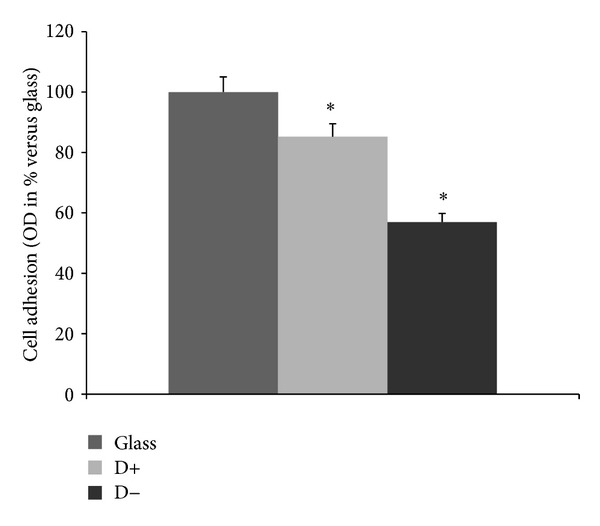
Adhesion rate of HUVECs. For MTT adhesion assay, the HUVECs were cultivated on glass, D−, and D+ for 48 h. The results for three independent experiments were expressed as percentage versus glass. *P < 0.05.

**Figure 9:**
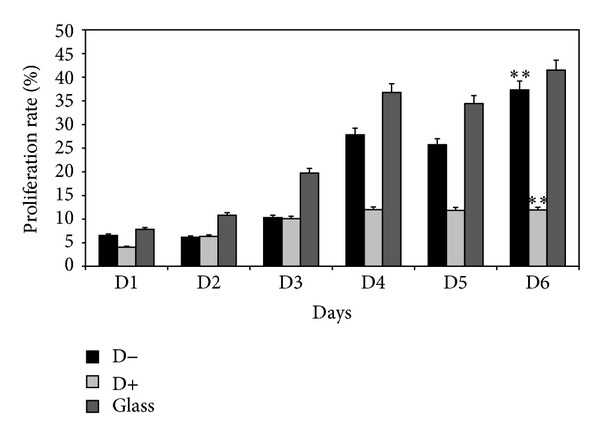
Proliferation rate (%) of HUVECs measured by cell counting. For proliferation cell counting assay, the HUVECs were cultivated on glass, D−, and D+ for 6 days. The results were obtained for three independent experiments. The percentage represents the number of cells that have proliferated compared to the initial number of seeded cells; *P* < 0.001.

**Figure 10:**
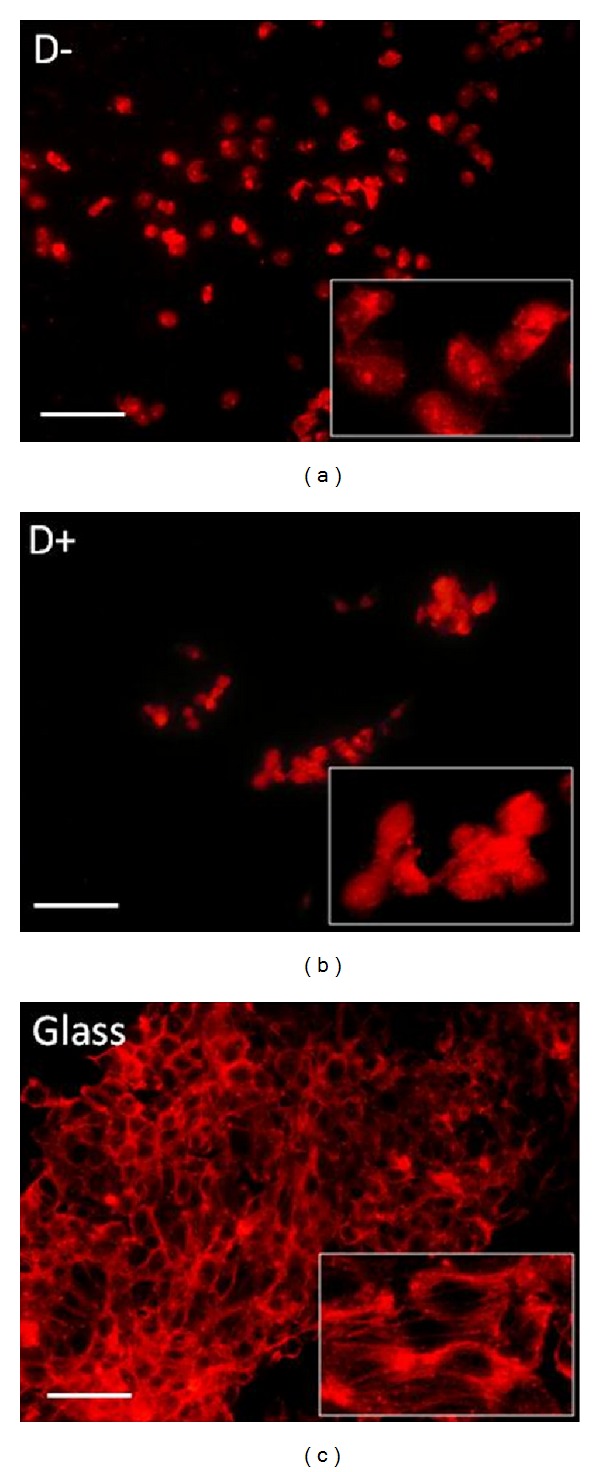
Cell response by fluorescence microscopy. For cell response, the HUVECs were cultivated on glass, D−, and D+ for 48 h and stained with Alexa Fluor 546 phalloidin. They were observed by fluorescence microscopy and photographed (red fluorescence of phalloidin, ×100 magnifications, bar = 30 *μ*m, with high-power view insets). Note in D− condition the well-spread cells with homogenous distribution of F-actin as compared to D+. Glass was the control.

In this part of the work, we analyzed HUVECs adhesion on D+, D− and glass surfaces (Figure 8). There was a significant difference of cells adhesion at 48 h observed between D+ and D− (*P* < 0.05). The results showed the significant decrease of cell adhesion on D− surface, as compared to D+. However, there was a higher cell proliferation at day 6 on D− surface, as compared to for D+, particularly from day 3 to day 6 (Figure 9, *P* < 0.001). In addition, the cell density was higher on D− than that on D+ (Figure 10). This reverse relationship is due to the fact that proliferation can be inhibited by too strong cell adhesion as seen in a previous study dealing with PEM for human gingival fibroblast response [26]. In addition, on D− surface the HUVECs were more spread and showed the homogenous distribution of F-actin (cytoskeleton) as compared to D+. Thus, if the PEM is terminated by negatively charged layer (which is in contact with the cells), this PEM film is ready to be tested* in vivo* to confirm the biocompatibility of the final D− coated stent implant. This difference could be attributed to Fn presence and its hydrophobic groups which are exhibited when no more proteins are adsorbing, as seen during DCA dynamical rinsing step. Indeed, endothelial cells are known to proliferate easily on hydrophobic surfaces. However, surface charge and roughness have also an important impact on cell response. In addition, the implantation of biomaterials into the* in vivo* models leads to exposure of the biomaterial surfaces to various proteins, the mix of many proteins, leading to much more complex conditions than those studied in this work. Any correlation between cell response and protein adsorption behavior should be proposed with precautions.

## 4. Conclusion

Dynamic contact angle (DCA) technique was used to analyze protein adsorption/desorption and reversibility on polyelectrolyte's multilayer (PEM) films based on dextran derivatives polymers. Glass was used as reference surface. Films were composed with diethylaminoethyl dextran (DEAE) as polycation (D+) and dextran sulphate (DS) as polyanion (D−). Fibronectin (Fn), vitronectin (Vn), and bovine serum albumin (BSA) and a mixture of the these proteins were studied during the adsorption/desorption processes. Wetting force was measured during wetting/dewetting, for 5 cycles in protein solution. Rinsing was evaluated just after 5 cycles of wetting/dewetting in PBS. Reorientation of protein conformation during rinsing phase was demonstrated, particularly for Fn on D− surface. HUVECs culture was performed on these PEM and compared to glass. Significant higher proliferation rate was evidenced for D− surface. Taken together, dextran sulphate was found to be a good candidate for coatings of the stents dedicated to biomaterial-based reendothelialization of vascular wall.
